# Review on the Production of Polysaccharide Aerogel Particles

**DOI:** 10.3390/ma11112144

**Published:** 2018-10-31

**Authors:** Kathirvel Ganesan, Tatiana Budtova, Lorenz Ratke, Pavel Gurikov, Victor Baudron, Imke Preibisch, Philipp Niemeyer, Irina Smirnova, Barbara Milow

**Affiliations:** 1 German Aerospace Center, Institute of Materials Research, Linder Hoehe, 51147 Cologne, Germany; lorenz.ratke@gmx.de (L.R.); philipp.niemeyer@dlr.de (P.N.); barbara.milow@dlr.de (B.M.); 2 MINES Paris Tech, PSL Research University, Center for Materials Forming (CEMEF), UMR CNRS 7635, CS 10207, 06904 Sophia Antipolis, France; tatiana.budtova@mines-paristech.fr; 3 Institute of Thermal Separation Processes, Hamburg University of Technology, Eißendorfer Straße 38, 21073 Hamburg, Germany; pavel.gurikov@tuhh.de (P.G.); victor.baudron@tuhh.de (V.B.); imke.preibisch@tuhh.de (I.P.); irina.smirnova@tuhh.de (I.S.)

**Keywords:** polysaccharide, droplets, particles, gel, drying, aerogel, mesoporous

## Abstract

A detailed study of the production of polysaccharide aerogel (bio-aerogel) particles from lab to pilot scale is surveyed in this article. An introduction to various droplets techniques available in the market is given and compared with the lab scale production of droplets using pipettes and syringes. An overview of the mechanisms of gelation of polysaccharide solutions together with non-solvent induced phase separation option is then discussed in the view of making wet particles. The main steps of particle recovery and solvent exchange are briefly described in order to pass through the final drying process. Various drying processes are overviewed and the importance of supercritical drying is highlighted. In addition, we present the characterization techniques to analyse the morphology and properties of the aerogels. The case studies of bio-aerogel (agar, alginate, cellulose, chitin, κ-carrageenan, pectin and starch) particles are reviewed. Potential applications of polysaccharide aerogel particles are briefly given. Finally, the conclusions summarize the prospects of the potential scale-up methods for producing bio-aerogel particles.

## 1. Introduction

In 1931, S. S. Kistler discovered highly porous materials by replacing the liquid in a gel with a gas in its supercritical state [1]. The latter allowed reducing shrinkage and solid network deformation during drying, keeping open porosity and preserving the structure of the gel. Kistler coined the term “aerogel” for these porous materials. Although almost forgotten for around four decades, the interest in aerogels became vivid again after the development of the alkoxide route to prepare oxide aerogels in the early seventies of the last century by Teichner [2]. In the last two decades new types of aerogels were developed, based on polyurethane [3,4], polyimide [5,6,7], polyamide [8], and hybrids of these with, for example, silica [9,10]. A new development took concurrently place: biopolymer aerogels or bio-aerogels, were synthesized from polysaccharides and proteins. Although monoliths of these aerogels were often studied, it became clear in the last years that particles or beads of polysaccharide aerogels are very promising for many applications in the areas of medicine, pharma and food industry and even as thermal insulating materials. This review is intended to give a brief overview on methods to produce polysaccharide aerogel particles, their microstructure and properties.

Before we start, let us define more precisely the term “aerogel,” as we use it here. The usage of this term is not standardized and therefore many porous materials are called “aerogels,” which can also be termed “open porous foams.” Aerogels differ from other porous materials. The existing definitions in the literature [11,12,13,14] can be combined and modified to provide a full description of these materials and their properties: An aerogel is an open porous, non-fluid/solid colloidal or polymeric network and therefore exhibits a low density and a high specific surface area. Open porous foams can be termed aerogel if the pores are also in the mesopores range, according to the IUPAC definition of mesoporosity.

In recent years, the research and development of the variety of aerogels production is increasingly growing due to their interesting physical and chemical properties such as sorption capabilities, thermal, mechanical, acoustical and optical properties. However, only few of aerogel types are commercialized so far [14,15]. There are many review articles reporting on aerogels structure and properties but mainly focusing on the inorganic and synthetic polymer ones [16,17,18], with a limited discussion on bio-aerogels [19,20,21,22,23,24].

Since 1931, after the invention of aerogels, metal oxide and synthetic polymer aerogels are well-developed with good understanding of the chemistry of sol-gel syntheses and their correlation with aerogel structure and properties [15,18]. Bio-aerogels are very “young” materials; their systematic synthesis started at the beginning of the 21st century. The correlations between the type of polysaccharide, synthesis pathways and resulting aerogel structure and properties are still far from being understood. Biopolymer molecules with their strong inter- and intramolecular forces, either physical or chemical interactions, tend to self-assemble on a molecular level producing entangled or crosslinked polymeric networks. It results in the formation of microstructure with interconnected nanofibrillar network. The essential difference between bio-aerogels and other organic (resorcin-formaldehyde, melamin-formaldehyde) or inorganic (silica, alumina, titania and others) ones is that the conventional aerogel synthesis starts with monomers, which react to form polymers and polymeric particles most often by hydrolysis and/or polycondensation mechanisms whereas the preparation of bio-aerogels starts with the dissolution of a polymer followed by solution gelation during which polymer chains rearrange themselves into an open porous network.

**Figure 1:**
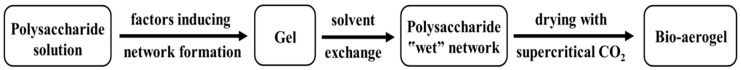
General pathway of synthesis of bio-aerogels from polysaccharide solutions via several steps, including a gelation step, an exchange of the solvent fluid and eventually supercritical drying with carbon dioxide.

The first attempt of bio-aerogels synthesis was reported by S. S. Kistler [1,25]. After some trials [26,27,28], the earliest publication leading to a bio-aerogel was reported in 1988 [29] describing the successful preparation of open porous cellulose particles from cellulose xanthate (viscose process). Only in the last two decades, scientists began to systematically investigate biopolymer aerogels from multiple sources such as agar, agarose, alginate, cellulose, chitin, chitosan, lignin, pectin, proteins and starch. Figure 1 illustrates the general pathway of synthesis of bio-aerogels. Interest in research, development and applications of the bio-aerogels with intriguing properties is rapidly growing [19,30,31,32,33,34,35,36,37,38,39,40,41,42,43,44,45,46,47,48,49,50,51,52,53,54,55,56,57,58,59,60,61,62,63,64,65,66,67,68,69]. Mostly, they are used as carriers, catalysts and supporting or template materials. Journal papers and reports on bio-aerogels describe their potential use for thermal insulation [43,44,45,46,47,48,49,50,51], drug delivery systems [19,53], tissue engineering and regenerative medicine [52], as catalysts [65,66,67,68,69] and sensors [54,55], adsorbents [56,57,58] and as well as raw materials for carbon aerogels [59,60,61] and organic-inorganic composite aerogels [51,54,62,63].

Perceiving potential applications of bio-aerogels in different emerging areas, it is important to look for their development on an industrial scale. Although the production of bio-aerogels in the lab scale is reasonably well mastered and a wide gamut of methods is developed, it is a great challenge to understand how to move from laboratory to industrial scale. Industries tend to adapt the very best available methods and technologies with cost being the major concern. The production of biopolymer-based aerogels in the form of particles has a tremendous advantage reducing significantly the process cost and time, especially with respect of the time needed for solvent exchange and supercritical drying [14].

Bio-aerogel particles are today not produced even on pilot plant scale. The general way of bio-aerogel production in particle form involves: droplet production, gelation, particle recovery, solvent exchange and drying in supercritical conditions. In this review, the possible production methods of bio-aerogel particles are discussed especially in view of their potential to be realized on a larger scale. First, general methods used for making droplets will be overviewed. Then the transitions from polysaccharide solution to a gel particle will be described followed by drying methods leading to porous structures. The sections mentioned above will give only brief overviews as far as literature on these topics is abundant. Finally, structure, properties and production of bio-aerogel particles will be presented and discussed in detail.

## 2. Engineering the Production of Droplets

Engineering the production of polysaccharide droplets from polysaccharide solutions had already been developed from laboratory experiments to industrial scale. The main objective of different methods is the ability to control droplet size, their size distribution, shape and morphology. With well-known particles’ production techniques, making polysaccharide droplets can be distinguished into two main methods:

(a) Formation of droplets in a gaseous phase with subsequent fall in a bath that induces gelation.
(b) Formation of droplets in a liquid phase which is immiscible with the biopolymer solution and on mixing leads to an emulsion.

In both cases, several parameters determine size and shape of the liquid droplets: the viscosity of each phase, the surface tension of the polysaccharide solution with respect to the surrounding medium (gas or liquid) and the dynamic interactions of the droplets with the matrix fluid (laminar or turbulent flow). In case (a) surface tension between droplet and air is essential when the liquid comes out of a nozzle while in case (b) the liquid is disintegrated in an immiscible fluid system into droplets with additional control of the interfacial tension between dispersed and continuous phases by surface active agents (surfactants). The main techniques for droplet production are described below.

### 2.1. Formation of Droplets in Gaseous Phase

#### 2.1.1. Conventional Dropping Method

**Figure 2:**
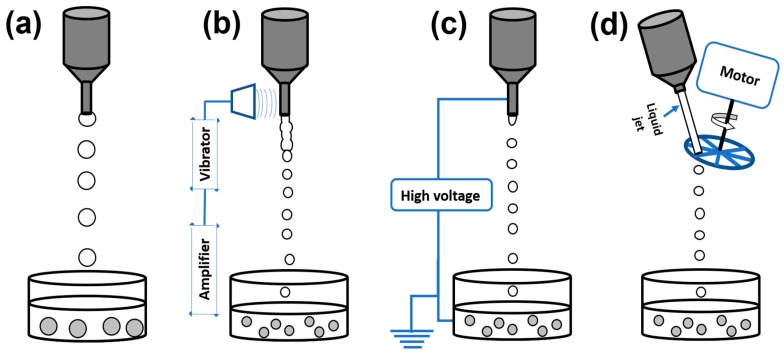
Illustration of dropping devices: (**a**) conventional dropping method influenced by gravity, surface tension and viscosity; breaking up of liquid jets into droplets stimulated by (**b**) vibrating nozzle method, (**c**) electrostatic forces and (**d**) a mechanical cutting device.

The droplets producing devices used mostly in the laboratory scale are tubes containing sharp tips at one end where droplets are produced and wide mouth on the other side where the liquids are filled, for example, pipettes and syringes (Figure 2a). In these dropping devices, the produced droplets at the sharp tip (orifice) continue falling freely under the influence of gravity into gelation or coagulation bath. As gravity is the only driving force to generate the droplet from orifice, these devices produce big droplets of the size of few millimetres, which is usually bigger than the nozzle diameter. The viscosity of the liquid and the nozzle diameter are important parameters defining the droplet size. In the conventional dropping methods the shape and size of the particles are dependent on the thermophysical properties of the solution such as surface tension, density and dynamic viscosity and on process parameters such as nozzle geometry and gravitational force [70]. With these dropping devices, the production capacity is very low which a serious disadvantage is. Multiplying the number of nozzles may improve the droplets production capacity on the laboratory scale. In some devices, the co-axial gas flow around the nozzle, but not spraying, was used to improve the speed of particles disintegration from the orifice [71]. In fact, the particles size becomes smaller in comparison with conventional dropping method as the gas induces shear at the nozzle tip and it is gently pushing the liquids out of the orifice, supporting the gravitational force. This method is considered to be spraying the droplets when the droplets size becomes smaller than 1 mm.

Currently the improved dropping devices producing monodispersed droplets and present on the market may be classified into the following groups according to the course of action breaking up the liquid jet: vibrating nozzle, electrostatic and mechanical cutting method (Figure 2b–d).

#### 2.1.2. Vibrating Nozzle Method

In this method, the monodispersed droplets are formed from a laminar liquid jet by applying superimposed vibrations with an optimum frequency either on the nozzle or on the liquid which is approaching the nozzle. These vibrations can be generated using sound waves [72,73]. Polysaccharide solution is pressurized using a pump or gas through a nozzle in order to produce a liquid jet. The superimposed vibrations destabilize the liquid jet (Rayleigh instability) and then the jet is disintegrated into monodispersed liquid droplets. This technique is also known as the prilling method in the literature [73,74]. In order to scale up the production of droplets, some companies (Nisco Engineering AG (Zurich, Switzerland) and Brace GmbH (Karlstein am Main, Germany) provide this technique with a multi-nozzle system. The major drawback of this technique is that this process can work only for solution viscosity lower than few hundreds of mPa.s [71]. The droplet size is estimated to be twice the diameter of the nozzle inner diameter and can be varied by changing the flow rate of the liquid and nozzle diameter [72].

#### 2.1.3. Electrostatic Method

In this method, the formation of droplets is enhanced by an electric field when the polysaccharide solution is extruded through a charged nozzle. The electric field pulls the liquid as droplets from the outlet of the orifice. The surface of the droplets of polysaccharide solution gets an induced electrostatic charge when it is disrupted from the orifice. The electrostatic repulsion of liquid droplets in gas phase prevents coalescence. The disintegration of the droplets and their size depends on the factors such as solution viscosity, nozzle diameter, distance from the collecting bath and applied voltage. For example, droplet size can be limited to few tens of micrometres by decreasing the nozzle diameter, reducing the distance between the electrodes and increasing the applied voltage [75,76]. The high processing capacity can be achieved using a multi-nozzle system [75,76]. This method of droplet formation is similar to the electrostatic atomization. But unlike electrostatic atomization which uses significant gauge pressure, the liquid droplets are produced from one low-velocity jet at the outlet orifice of a narrow needle. In this method, the produced droplets have almost the same size. The applied electrical potential can be a static or pulsed one [77]. This method is limited to low viscosity liquids.

**Figure 3:**
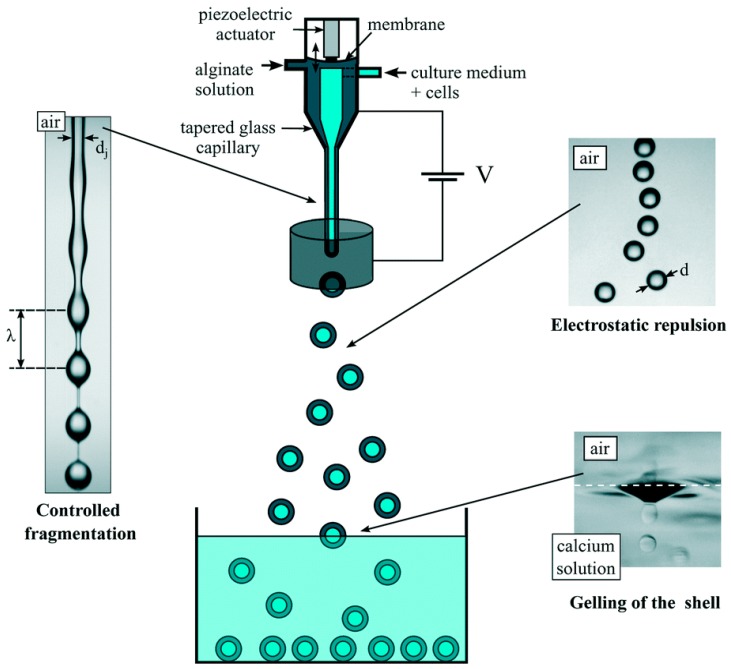
Schematic workflow of the generation of capsules containing liquids; reproduced with permission from reference [78].

There are dropping devices with electrostatic acceleration of drop formation which allow to supply two immiscible liquids at the same time and thus produce micro capsules directly at the outlet orifice [78,79]. A schematic drawing of such device is shown in Figure 3.

#### 2.1.4. Mechanical Cutting Method

The preparation of particles with a size in the range between the few hundreds of micrometres and a few millimetres with a high productivity and an economic efficiency is possible using the JetCutter technology developed by Vorlop and Berford in 1996 at geniaLab^®^ [80]. The essence of technology is based on the mechanical cutting of a continuous liquid jet. The polysaccharide solution is pressurized through a nozzle with high velocity to obtain a stable liquid jet. A rotating tool with strings/wires cuts a liquid jet into cylinders of equal size which become almost spherical droplets during their flight in air while falling down into the gelation bath. This JetCutter technique allows to prepare spherical particles with a predetermined size by varying the parameters like cutting frequency, jet speed and nozzle diameter. Compared to the classical dropping method this method has a high production rate and allows to work with highly viscous liquids, dispersions and melts of preferably shear thinning behaviour (viscosities between 0.2 Pa.s and 110 Pa.s) [81,82].

**Figure 4:**
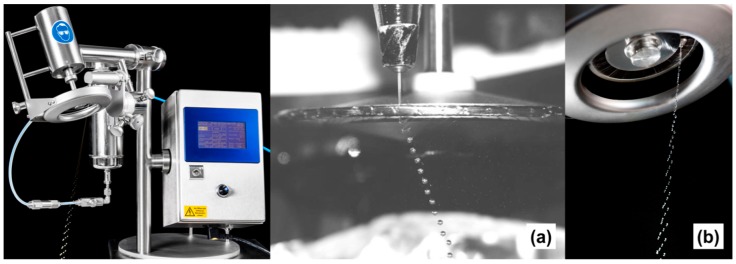
Image showing the table top JetCutter machine on the left and different Jet cutting tools producing a single stream of droplets; image reproduced with permission from [83] (**a**) and multi-stream of droplets (**b**).

JetCutter technique is very favourable to produce particles in the range between 0.2 and 0.8 mm. Two factors influence the productivity: (1) the rotating frequency of the spinning and cutting wheel which determines the droplet generation rate and (2) the initial droplet diameter. Figure 4 shows that a stream of droplets production can be modified from single- to multi-stream by varying the design of cutting discs. Depending on the desired throughput, different installations can be carried out such as multi-nozzle system, designing the cutting wires and multiunit installations [83].

A disadvantageous feature of the JetCutter technology is material loss, which stems from so called cutting and spraying losses. While spraying losses can be minimized, cutting losses cannot be minimized by modification of some cutting parameters like the inclination of the cutting disk and/or the thickness of the cutting wires. Inclination angle depends on a liquid’s flow rate and a frequency of cutter’s rotation. Prüsse et al. [84,85] mathematically described the process parameters in order to use the JetCutter process in an efficient manner.

#### 2.1.5. Spraying/Atomization

**Figure 5:**
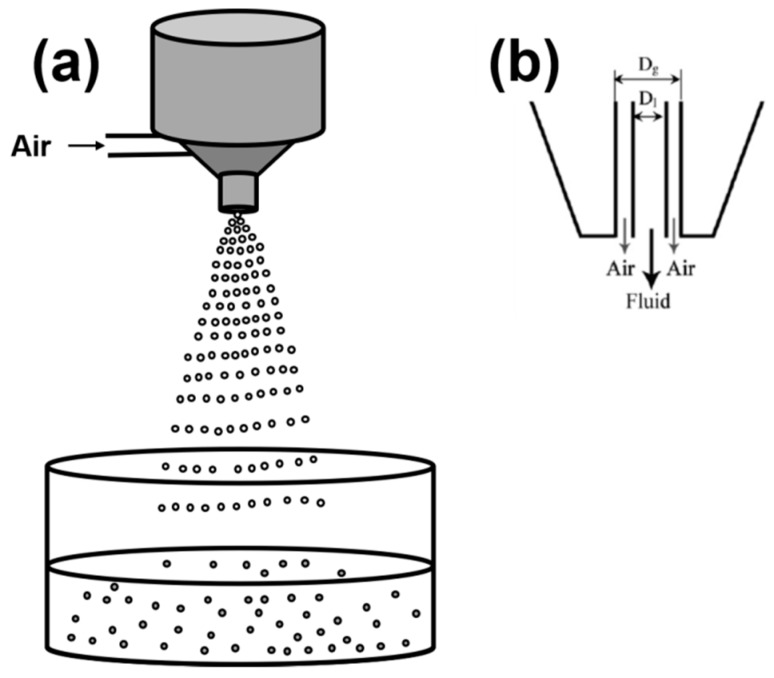
Scheme showing atomization of a solution (**a**). The atomizer nozzle (**b**) showing one of the example used for the production of κ-carrageenan gel particles using atomizing technique. Dl is the diameter of the fluid nozzle exit and Dg is the diameter of the gas nozzle exit; reproduced with permission from reference [86].

Atomization is a process of full disintegration of a stream or jet of an incompressible liquid leading to the formation of poly- or monodispersed droplets in a gas phase or in vacuum, which is achieved by atomizer nozzles. Figure 5a shows the stream of atomized droplets falling in the gelation bath. In Figure 5b, the schematic diagram of one of the kind of atomizer nozzle is shown which was used for the production of κ-carrageenan gel particles [86].

Lord Rayleigh developed more than 100 years ago a first hydrodynamic model being able to describe the break-up of a liquid jet into droplets, which was further developed by many authors [87,88]. Basically, atomization is a result of the competition between several factors with one of them being: surface tension. The surface energy of a cylindrical jet can be reduced, if the liquid jet is transformed into droplets of a certain size given by the Plateau-Rayleigh instability criterion for Newtonian liquids. Second viscosity affects the break-up process, especially in non-Newtonian liquids. In most cases, turbulent flow of the liquid jet, cavitation inside the nozzle, and aerodynamic interaction with the surrounding gaseous medium contribute to atomization. Thus, atomization is a multi-stage process and the production of the droplets depends on many operating parameters. Once droplets occur after initial breakup of the liquid jet or sheet, the droplets themselves can be unstable and further disintegrate into smaller ones. This process of self-disintegration of droplets can continue leading to a cascade of finer and finer droplets. In this way, the droplet size characteristics of a spray are not governed simply by the initial droplet sizes but mainly by the extent to which the largest of these drops are further disintegrated during secondary atomization. Models have been developed to predict the drops size spectrum and these equations can be applied to determine the volumetric bead diameters for atomization processes with different types of nozzles [87,88,89,90].

Several atomizing devices are currently available. They may be divided into groups: Pressure Jet Atomization, Fan Spray Atomization, Twin-fluid Atomization, Rotary Atomization, Effervescent Atomization, Electrostatic Atomization, Vibration Atomization and Whistle Atomization. The fundamentals of different atomization process can be found in the literature [91,92,93]. All these atomizing units are widely used in the pilot scale production of wet spherical particles. Very recently it has been applied for open porous particles production [94,95].

### 2.2. Formation of Liquid Droplets in Oil Phase

**Figure 6:**
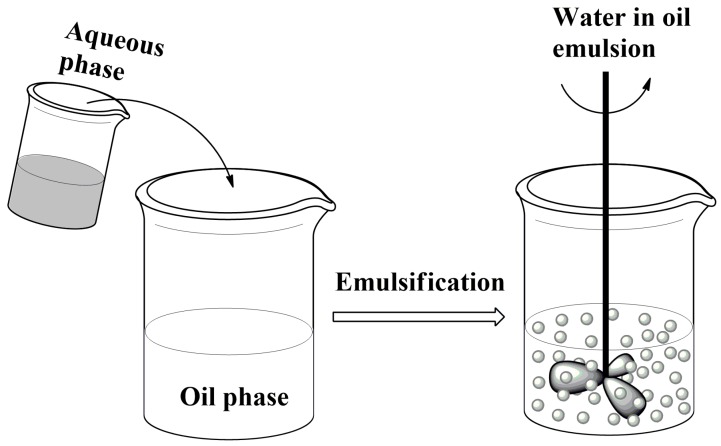
Schematic representation of emulsification process: polysaccharide solution (aqueous phase) is dispersed in oil phase (water-in-oil emulsion) followed by gelation of each individual droplet.

Emulsification is a process in which the dispersion of two immiscible liquids is stabilized by an emulsifier which is often a surfactant. In this process, fluid may be shaped into spherical droplets in an immiscible liquid phase. Continuous energy input such as stirring or agitation and adding suitable surfactants keep the dispersed phase in the continuous phase as it is a dynamic process (Figure 6). Once a stable emulsion is formed (for example, “water-in-oil,” w/o), chemical and/or physical impact may trigger gelation and further stabilizes the droplets.

Water-to-oil ratio is one of the parameters influencing emulsification. This ratio usually ranges from 1:2 to 1:10 at the lab scale. A transformation from lab to large scale production poses evidently some questions like the optimal ratio as well as the oil recovery. Roughly, the ratio of the viscosities of the aqueous to the oil phase needs to be below 1 in order to break the droplets. The surfactant concentration and its hydrophilic-lipophilic balance (HLB) value should also be taken into account to stabilize the emulsion droplets. The most common recommendation for w/o emulsions is to keep HLB value in the range of 3–6 [96].

This method of droplet production is often employed for polysaccharides soluble in the aqueous phase. A stable emulsion and a steady average droplet size can be obtained within few minutes, depending on various process parameters [97]. Poncelet et al. [98] has shown in the production of alginate particles that the droplet size can be directly affected by the apparatus geometry. With all other conditions kept constant, an increase of the mixing rate from 200 to 500 rpm leads to a decrease in the particle size by a factor of 5.1 for a turbine with baffles, whereas only by a factor of 2.7 for marine impeller.

To achieve the scale-up from lab-scale to pilot and industrial scale, several approaches should be considered depending on the emulsion-gelation system. First of all, the viscosities of the continuous and dispersed phases and of the emulsion itself (that is usually higher than that predicted by the mixing rule) are very important. For the systems where the viscosities are relatively low, standard scale-up strategies for stirred tank can be applied. Depending on the flow regime (laminar or turbulent), the scale up can be performed keeping similar shear stresses (for laminar regime) or energy dissipation rate (for turbulent regime) [99].

It should be noted that as the volume of a stirred tank increases, the trigger used to induce the gelation in emulsion (heat, oil soluble or insoluble chemicals) can take a significant amount of time required to reach the entire volume of the dispersed phase. If the same energy input (stirring intensity) is used during the gelation step as during the emulsification, it is possible for gelled or gelling droplet to be sheared thus losing their spherical shape [100]. If the stirring intensity is lowered to avoid such problems, the non-gelled droplets have a chance to coalesce. To avoid such inhomogeneous gelation, the gelation can be carried in-line, where the emulsion would be discharged in another vessel and the gelation trigger added at the same time, allowing for better local homogeneity of the gelling system.

For systems that present relatively high viscosity, the appearance of dead volumes in stirred tank (fraction of the fluid presenting no or low flow) impedes the emulsification process and favours coalescence (thus reducing the fraction of the smallest droplets). In this case, the use of continuous emulsification devices such as in-line toothed gear rotor stator machine or colloid mill could be an alternative and is currently under investigation. Such emulsification devices are widely used in the food industry [101], illustrating the validity of such approach. In this case the gelation could also be induced in-line resulting in a fully continuous emulsion-gelation process.

## 3. Gelation of Polysaccharides Droplets to Produce Gel Particles

**Figure 7:**
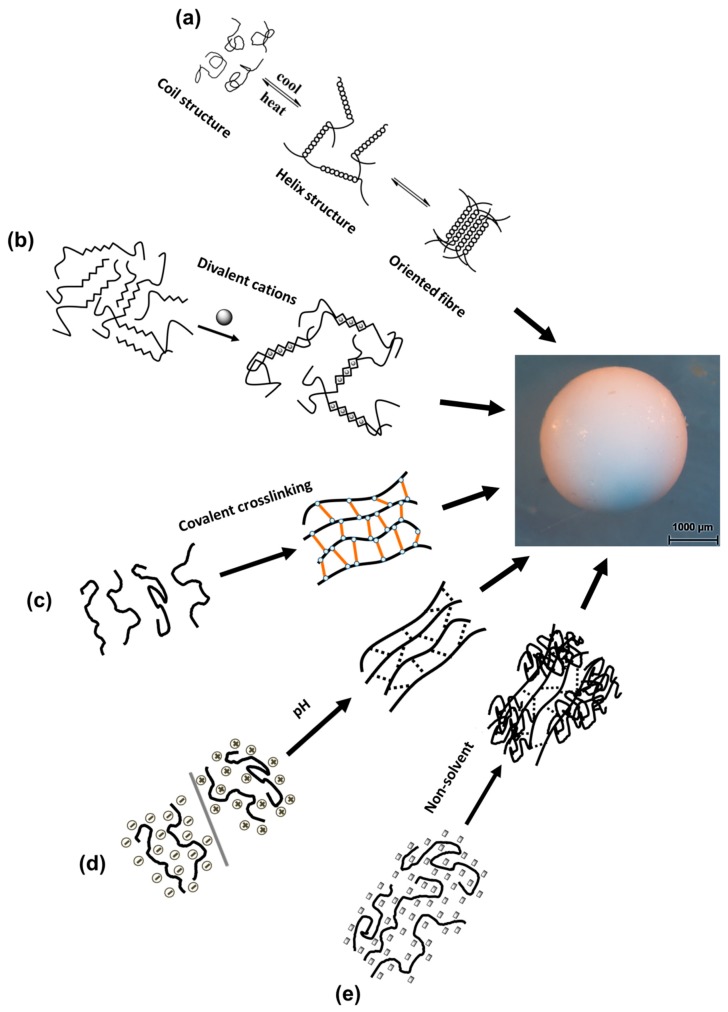
Illustration of the main mechanisms of formation of polysaccharide gel particles: (**a**) temperature-induced (thermotropic) gelation in which the polysaccharides undergo structural transition from coil to helix and then to double helix, (**b**) ions-induced (ionotropic) gelation in which the polysaccharide molecules are crosslinked by ions, (**c**) covalent crosslinking approach in which the polysaccharide chains are covalently crosslinked to form gel network, (**d**) pH-induced gelation and (**e**) non-solvent approach to produce a non-solvent filled gel network.

The gelation of polysaccharide droplets can be classified in a variety of ways and here we use “gelation” in the sense of “formation of a network.” Among them are the factors inducing the structural association of molecules such as: temperature (thermotropic; cryogelation), chemical crosslinking (ionotropic; chemical modification), pH (changing pH of the solvent medium) and non-solvent induced phase separation (coagulation). There are many review articles discussing the gelation mechanism of polysaccharides [102,103,104,105,106,107,108,109]. Figure 7 illustrates the gelation methods of polysaccharides under different conditions, showing a gelled particle as an example. So far reported in the literature, bio-aerogels in particle form have been successfully prepared using agar, alginate, cellulose, chitin, chitosan, κ-carrageenan, pectin and starch. The case studies of these polysaccharides’ gelation in the view of the production of aerogel particles are briefly overviewed below.

### 3.1. Temperature-Induced Gelation

Temperature-induced gelation is also called thermotropic gelation. In this process, polysaccharide molecules associate themselves often into oriented form in response to temperature, usually upon cooling (for example, agarose, κ- and ι-carrageenan). In the gelation mechanism of agarose and κ- and ι-carrageenan, the polysaccharides undergo reversible structural transitions from helix to random coil structure and vice versa upon heating and cooling of their solution (see Figure 7a). The association of these helices leads to double helix formation, then proceeding to a gel network. In carrageenans helices are stabilized by added metal ions while in agar helices are stabilized by hydrogen bonds.

Starch also undergoes thermal gelation but the mechanism is different from that of agar and carrageenans. Starch is a combination of two polymers: amylose (linear) and amylopectin (branched). When placed in water and upon heating, starch granules swell, lose their crystallinity and amylose is leaching out. Gelatinization temperature, corresponding to the loss of granule integrity and melting of amylopectin, strongly depends on starch source. Further cooling leads to molecules re-association and formation of opaque gels; this process is not reversible and called retrogradation. Amylopectin may re-crystallize thus increasing the rigidity of gels. The principles of starch gelatinization and retrogradation mechanism are discussed in the literature [106,107]. The gelation process and gel properties are highly depending upon starch source, composition, concentration, crystallinity and processing conditions.

Contrary to agar, starch and κ-carrageenan solutions, cellulose dissolved in 7–9% NaOH in water are gelling with temperature increase, and also with time [110]. The thermodynamic quality of this aqueous sodium hydroxide solvent decreases with increasing temperature preferentially leading to cellulose-cellulose inter- and intramolecular (and not cellulose-solvent) interactions. As a result, cellulose coils contract in dilute state and solutions are gelling when being above polymer overlap concentration. The additives such as urea [111], thiourea and ZnO [112] delay gelation but the mechanism remains the same [113]. Cellulose gel can also be produced by cryogelation method. It was recently reported that crosslinked cellulose nanofibrils are sprayed in liquid nitrogen to produce frozen cellulose gel particles [94,95,114].

### 3.2. Crosslinking-Induced Gelation

#### 3.2.1. Ions Crosslinking-Induced Gelation

In this process, the polysaccharides are crosslinked and form a gel network in the presence of ions. This process is also called ionotropic gelation. Alginate, pectin, κ-carrageenan and chitosan are polyelectrolytes having active functional groups, such as carboxylate, sulphate and amine which are involved in this gelation process. Alginate and pectin form gels in the presence of divalent cations. More in detail, divalent (often calcium) cations bind carboxylate functional groups present on polymer backbone (1,4-linked-α-L-guluronic acid in the case of alginate and α-linked anhydrogalacturonic acid in the case of pectin) forming the “egg-box” structures resulting in a gel (Figure 7b). For pectins, calcium-induced gelation is more pronounced for lower-methylated pectins and at pH around 3–3.5 as it depends on the degree of esterification.

Sulphate functional groups of κ-carrageenan, in its coil structure, bind to the specific cations (such as potassium, rubidium or caesium) forming a hard gel. Gelation temperature depends on the concentration of these specific cations. The co-ion of the specific cation, i.e., the specific anion can act as potent helix stabilizer. Specific ions bind to the different classes of sites on the helix as revealed by NMR experiments and applying the Poisson-Boltzmann model [115,116,117,118].

Chitosan with its amine functional group can undergo ionotropic gelation when anionic counter ions such as tripolyphosphate, sulphate and citrate present in the solution. The formation of electrostatic interactions, existing between cationic chitosan and anionic counter ions promotes gel network formation [119,120,121].

Two gelling polymers can also be mixed to form an interpenetrated network. For example, pectin and alginate can be intermixed in the presence of calcium ions leading to “mixed gels.” The gel network consists of heterogeneous interactions between two polymers where L-guluronate and D-galacturonate bound by calcium ions [103,122].

#### 3.2.2. Covalent Crosslinking-Induced Gelation

Gels can be formed via covalent cross-linking which leads to irreversible chemical networks. However, many of cross-linking agents are not biocompatible and thus are not often used to cross-link polysaccharides, as far as the latter are mainly used for food, feed, cosmetics and pharma.

An example of a cross-linking agent for cellulose is epichlorohydrin (ECH). Both cellulose and ECH are dissolved in NaOH-water, mixed and gelation occurs in time via C2 hydroxyl groups. Cellulose cross-linked with ECH and swollen in water after washing out NaOH has the highest degree of swelling at anhydroglucose:ECH stoichiometric ratio and reaches 3000–4000 wt % [123]. Another example is compounds that chemically cross-link cellulose in aqueous medium, they are used to reduce the fibrillation of Lyocell fibres [124] (i.e., mild cross-linking of the surface layer of never-dried fibres) are: dichlorohydroxytriazine, 1,3,5-triacryloylhexahydrotriazine, 2,4-diacrylamidobenzenesulphonic acid, N-methylol resins and dialdehydes. 

Chitosan microspheres are produced by mixing chitosan and glutaraldehyde solutions in oil containing a surfactant [125,126,127,128,129]. Here, chitosan chains are covalently cross-linked by the glutaraldehyde molecule (see Figure 7c). This is a Schiff base reaction between amine and aldehyde. In the same way, other aldehydes such as glyoxal and formaldehyde are used for crosslinking chitosan chains [130,131].

To vary the mechanical properties of alginate gels various cross-linkers were used like adipic dihydrazide, lysine, and poly(ethylene glycol)-diamines [132]. The type of cross-linking molecule and the cross-linking density determines both the mechanical properties and the degree of swelling in alginate hydrogels.

### 3.3. pH-Induced Gelation

The dissolved polysaccharides under alkaline or acidic condition can undergo gelation by changing pH of the solvent medium. At the contact point of the acidic or alkaline solution and a droplet of polysaccharide solution, the gelation starts to occur and immediately forms a shell. Then the diffusion of ions through the shell promotes the complete gelation (Figure 7d). Usually a high concentration of acidic or alkaline condition is maintained in the regeneration bath to ensure the complete diffusion of ions and regeneration of polysaccharides. Chitosan, pectin and alginic acid gel particles are mainly prepared by this method.

At low pH, the gelation of alginate and pectin solution is due to intermolecular hydrogen bonding between protonated groups (carboxyl, hydroxyl). In the case of pectin, gelation is also stabilized by hydrophobic interactions of methylated groups. Increasing the pH leads to deprotonation of acidic groups which prevents aggregation of chains and eventually gelation. For example, alginic acid gels can be prepared by protonating the sodium salt of carboxylate functional groups in acidic solution [133,134]. Herein the sodium salt of carboxylate functional groups becomes carboxylic acid promoting the hydrogen bonding and network formation. It should be kept in mind that polymer degradation may occur in solutions at pH ≤ 1.

Chitosan gel particles can be prepared at higher pH value. Chitosan can be dissolved under mild acidic condition by protonating the amine functional group, usually using acetic acid and then gel particles are produced under alkaline medium (NaOH solution) [67,133,134,135,136,137,138]. The pH value of the alkaline solution was maintained above the pKa value (6.3) of –NH_2_ functional groups in order to deprotonate the amine functional groups.

### 3.4. Non-Solvent-Induced Phase Separation

Another way of making gel-like stable “wet” particles or “monoliths” from polysaccharides is non-solvent induced phase separation. This is well known in preparation of membranes as “immersion precipitation.” It is usually applied to a non-gelled solution, in order to shape it and further proceed to drying. This approach is widely used in the processing of cellulose in fibre spinning and film casting. The principle is as follows: a non-solvent is added to a polysaccharide solution diffusing inside it. The solubility of the polymer decreases in as much as the non-solvent proportion increases. This is a diffusion initiated phase separation of the polymer solution into polymer-rich and polymer-lean regions [109]. The phase separation process was reported for making bio-aerogels monoliths and particles from cellulose dissolved in the ionic liquid, EmimAc (1-ethyl-3-methylimidazolium acetate) [139,140,141], in a hot solution of cellulose in NMMO (N-methyl morpholine-N-oxide monohydrate) [32], in cellulose dissolved in NaOH [142], as well as in alginate [109] and pectin [143] solutions. Sometimes these materials are called “hydrogels” if non-solvent is water (case of cellulose) or “alcogels” if non-solvent is ethanol (case of most polysaccharides).

The process of non-solvent induced coagulation allows making particles if polysaccharide solution is dropped (with a syringe or via atomisation) in a non-solvent bath. An interesting feature here is that polysaccharide macromolecules are shrinking upon the addition of non-solvent, but not completely collapsing if polymer concentration is above the overlap concentration. Polymer chains are self-associating and forming a 3D self-standing network with non-solvent in the pores (see Figure 7e). The nature of non-solvent and polysaccharide solution viscosity (which is dependent on polymer molecular weight and concentration) play an important role in sample shrinkage and final aerogel density and morphology. 

Cellulose particles were obtained by coagulating cellulose from solution in aqueous NaOH [142,144,145,146,147] (before gelation occurred) and in ionic liquids [148,149]. A similar approach but applied to emulsions was reported in reference [150]: cellulose wet beads were obtained via emulsification of cellulose-NaOH-urea-water solution with paraffin oil and Span-80 followed by separation and coagulation of cellulose in aqueous medium. Usually strong acidic solutions are employed to coagulate cellulose: H_2_SO_4_ [151], HNO_3_ [146], HCl [144,150,152]. In this case, aqueous strong acidic medium is considered as non-solvent due to coagulation of cellulose and the gel-like structure is induced by change in pH of the medium. 

Chitin gel particles were prepared with a same approach by dissolving chitin in an ionic liquid (1-butyl-3-methylimidazolium acetate) and extruding the solution through a nozzle into a coagulating bath containing ethanol [153]. In another approach, polymer-grafted chitin polymer, chitin-g-poly(4-vinylpyridine) particles were prepared by dropping the DMAc-LiCl solution into a coagulation bath containing ethanol [154].

A combination of ionic gelation and non-solvent induced phase separation was reported to make alginate/pectin aerogel particles [74]. The core(pectin)-shell(alginate) particles were made with the prilling technique. The capsules were dropped in ethanol bath containing CaCl_2_ and further dried with supercritical CO_2_. In this case pectin cross-linking with calcium ions occurred at the same time as polymer coagulation.

Another way to shape non-gelling polysaccharide solutions in a 3D form (especially those polysaccharides that are not easily soluble, such as cellulose and chitosan) is to perform chemical modification (or derivatisation) of the polymer, dissolve it, shape and regenerate (or de-derivatise). An example is the dissolution of a cellulose derivative like viscose, cellulose esters or ethers in a suitable solvent and then cellulose regeneration is achieved in a bath whose chemical composition is tuned to perform “de-derivatisation” (hydrolysis, deacetylation, etc.) [29,155,156,157]. Sometimes regeneration and shaping is performed in the same coagulation bath. For example, dissolving cellulose acetate in a water-miscible organic solvent like acetone or DMSO and dispersing this solution in a water bath leads to the formation of particles [157]. Cellulose can then be regenerated by hydrolysis.

## 4. Particle Recovery/Solvent Exchange

When polysaccharide gel particles are formed, they have to be separated from the continuous phase (either gelling bath, or coagulation bath, or oil). The particles are usually collected by filtration or centrifugation. Most of polysaccharides particles are produced in aqueous media until or unless the gelation happens in an organic solvent. The solvents, reagents and the additives, which are used for gelation and emulsion stabilization, have to be completely removed through several washings. Once the gel particles are confirmed to be pure, that means washing step is completed. If the gel particles contain water, the solvent exchange is necessary to undergo supercritical drying as far as water is not miscible with CO_2_. 

The exposure of polysaccharide gels to highly concentrated organic non-solvents (>30–50%) as it is done during a solvent exchange procedure leads, however, to a significant shrinkage and hence a change of the microstructure of the resulting aerogels. Therefore, it is crucial to maintain a balance between the amount of the organic non-solvent at the recovery step needed and the amount that wet gels can tolerate. Comparing monoliths to particles, it is fortunate that gel particles are less sensitive to the large concentration gradients during the solvent exchange. In the literature [35,74,133,134,158,159,160,161], the successive immersion of hydrogel particles in a series of alcohol-water concentration has been practiced. The interaction of the organic non-solvent with the gel matrix is a complex matter. The number of the organic non-solvents presently used for the solvent exchange is very limited, mainly alcohol and acetone being used as they are well miscible with CO_2_. However, principally all organic solvents, which are suitable for the later steps of supercritical drying (or any other drying type), can be applied. Thereby the interactions solvent-matrix can be quantified, for instance, with solubility parameters as demonstrated in reference [162]. Solubility parameters are widely used to predict the compatibility of polymers and affinities to surfaces to improve dispersion and adhesion. Even for polymers insoluble in a certain solvent the solubility parameters can be correlated with swelling and shrinkage. In case of alginates [162] a clear trend was observed for shrinkage behaviour of alginates in 16 different solvents: the alginate gels shrank less in the solvents with solubility parameters closer to the one of alginate. 

The kinetics of the solvent exchange and corresponding shrinkage of the gels can be modelled by the approaches commonly used in polymer science. For instance pseudo second order kinetics, normally used for the explanation of swelling kinetics, was successfully applied to describe the solvent exchange in alginate gels, what leads to a conclusion that it includes simultaneous adsorption and permeation processes [162]. Still comprehensive studies are required to clarify the interplay in the process parameters and different polysaccharides in order to minimize the shrinkage and to maintain the initial structure of the hydrogel. 

In the view of the scale up of the production process, different techniques can be applied to realize the solvent exchange on a larger scale. For example, polysaccharide particles can be pumped as aqueous slurry to the solvent exchange vessels, where the concentration gradient can be realized analogous to the chromatographic techniques. After the solvent exchange is completed, the particle slurry can be analogously transferred to the drying vessel.

## 5. Drying of Particles

Drying of wet gel is a process being the last and the most critical step in aerogel production. After solvent exchange, polysaccharide aerogel precursor, the wet gel, is a heterogeneous structure with a high porosity and pores filled with a liquid. The volumetric pore fraction of the liquid is generally more than 0.95. Removal of the pore liquid is termed drying. Since drying aims at preserving the pore volume of the matrix, it is necessary to minimize shrinkage of the solid network and prevent collapse during drying to obtain aerogels with properties desirable for a wide variety of applications.

Several drying processes, such as ambient, vacuum, freeze and supercritical drying are employed in preparing dried gels. In general, the drying process collapses the microstructure of the gel body to certain extent due to the surface tension that is created in the gel body between the solid-liquid-gas interfaces and, of course, capillary stress gradients stemming from the dispersion of pore sizes. It was recently reported for monolithic gel bodies that the drying processes strongly influenced the materials properties and the porous structures [140,163]. For polysaccharide wet gels, the ambient drying method provides highly aggregated microstructure due to massive shrinkage leading to densely packed solid with no porous structure [163]. However the porous structure may resist to collapse to a certain limit if the wet gel body has organic solvent molecules like alcohol with low surface tension and low vapour pressure [163]. The most probable reason is that biopolymers are intrinsically hydrophilic. Chemically modifying the hydrophilic –OH functional groups to hydrophobic environment can assist the ambient drying. Recently, low density, open porous and hydrophobic cellulose materials were prepared via ambient drying by chemically modifying the –OH functional groups with tritylchloride [164]. In this method, depending upon the degree of substitution, the chemical modification may lead to the development of unusual microstructure due to the different manner of self-assembly of cellulose molecules and lack of hydrogen bonding. 

In the case of freeze drying, the liquid in the gel body is frozen and sublimed under regulated vacuum. Usually this is done when the fluid in the network pores is water. The volume shrinkage can be limited to 40–50%. Unless special precautions are taken to prevent the growth of ice crystals, freezing may destroy the pore structure and damage the nanostructured gel body as freezing always implies the growth of crystals. Freezing is associated with the formation of a dendritic network of the crystalline solvent phase. The dendrites are, depending on the cooling rate, typically in the range of few up to a few tens of micrometres size; they push the walls of the network at the crystal boundaries and thus destroy the morphology formed during gelation [165]. Therefore, freeze drying usually leads to an open porous material with a pore size in the range of several micrometres. Although quite often termed aerogels in the literature, they are better termed open porous foams (or cryogels).

In supercritical drying, the pore liquid from aerogel precursor after solvent exchange can be extracted under supercritical condition. A fluid reaches its supercritical state when it is compressed and heated above its critical temperature and pressure. Supercritical fluids have liquid-like densities and gas-like viscosities [166]. Supercritical carbon dioxide is very attractive among other supercritical fluids and most employed in many industries as it has relatively easy accessible critical conditions, is nontoxic, environmentally friendly, widely available and cheap. Therefore, CO_2_ is used to extract organic solvent from the gel pores (this process can be actually classified as supercritical fluid-liquid extraction) and then vented out at constant temperature higher than its critical point. In this drying process, the gel network can be preserved without cracks as there are no capillary stresses. With the great advantage of this drying process, many industries produce commercial silica-based aerogel products in different forms, mostly sheets or panels. The extraction time depends mainly on the thickness of the samples. Therefore, it can be still reduced from the several hours needed for thick monoliths to only few minutes for polysaccharide particles of millimetre size. In general, the production time of aerogel particles is estimated to be a factor of 10 to 100 times shorter than for monolithic aerogels, since both the solvent exchange and the drying time are much shorter due to the smaller diffusion length and possibility to influence the mass transport by the suitable flow regime in the particle bed [14].

In the following chapter, aerogels produced from different biopolymers in the form of particles are discussed along with their properties and production methods.

## 6. Characterization of Bio-Aerogels

Methods used to characterize the structure and properties of classical aerogels can be applied for bio-aerogels with some precautions, as it will be mentioned below. The first property to report is bulk density ($\rho_{bulk}$); it is usually determined by simply measuring sample mass (weighing) and volume (dimensions). Another way is to use powder densitometer which allows measuring the volume of samples of geometrically complex shapes. Skeletal density of open pores materials is measured with helium pycnometer. The skeletal density $\rho_{skeletal}$ of polysaccharides is known to be 1.5–1.7 g/cm^3^.

The porosity ε of bio-aerogels can be then calculated from Equation (1) as follows:

(1) $$\varepsilon =\frac{V_{pores}}{V_{total}}=1-\frac{\rho_{bulk}}{\rho_{skeletal}}$$

Scanning electron microscopy (SEM) is a very useful tool to characterize aerogel morphology, however, image analysis software cannot be used to measure pore sizes because of irregular shapes of the pores. 3D tomography is also a way to visualize bio-aerogel morphology but it does not allow building pore size distribution below few hundred nanometres.

Specific surface area (*S_BET_*), pore volume (*V_pores_*) and pore size distribution are the main parameters characterizing aerogel texture. As well as for classical aerogels, *S_BET_* of bio-aerogels is determined using nitrogen adsorption technique and Brunauer–Emmett–Teller (BET) approach. 

The standard methods applied for measuring pore volume and pore size distribution in classical aerogels use Barrett-Joyner-Halenda (BJH) approach (for nitrogen adsorption) and/or mercury porosimetry. Bio-aerogels possess mesopores and also small, large and very large macropores (from several hundreds of nanometres up to several microns). Thus, the BJH approach, which considers only mesopores and small macropores, cannot be applied to characterize pore size distribution in bio-aerogels. It was demonstrated that BJH approach takes into account only 10–20% of the total pore volume in bio-aerogels [44,50,133,167]. Pore size distributions in bio-aerogels are thus clearly not limited to mesopores region. It may be possible that nitrogen condensation induces pores’ contraction [168] or bio-aerogels are simply compressed at higher nitrogen pressure. These limitations in BJH approach for bio-aerogels should be kept in mind in order to avoid artefacts and wrong understanding of bio-aerogel morphology. When mercury porosimetry is used, very often bio-aerogels are compressed: mercury is not penetrating in the pores [44,169].

The problem with the applicability of BJH approach on bio-aerogels is reflected in the “measured” pore volume: while the volume of mesopores, as obtained with BJH approach, is usually around 0.5–2.5 cm^3^/g, the total pore volume, calculated according to Equation (2), can reach several tens of cm^3^/g due to macroporosity [44,50,133]:

(2) $$V_{pores}=\frac{1}{\rho_{bulk}}-\frac{1}{\rho_{skeletal}}$$

Thermoporosimetry was used for the determination of pore size distribution [141,170]. This approach is based on the measurement of the experimental shift of the melting point of an interstitial liquid caused by its confinement in small pores. Differential scanning calorimeter is used for this purpose and the method was applied to characterize cellulose aerogels. This approach provided a good correlation with cellulose aerogel morphology seen by SEM; it also demonstrated a significant difference with pore sizes predicted by BJH method.

Several other features, specific for bio-aerogels, should also be taken into account in order not to obtain artefacts. Native polysaccharides are very sensitive to humidity (and several are hydrosoluble) and thus adsorb water vapours. Bulk density and thermal conductivity of “aged” bio-aerogels increase as compared to their corresponding values obtained on samples just after drying. A three to five fold increase of thermal conductivity of freeze-dried cellulose II was demonstrated when relative humidity increased from 0 to 60% [171]. It should be kept in mind that subsequent drying leads to pores’ irreversible closing, for cellulose it is known as the “hornification” phenomenon. Pore closing leads to aerogel shrinkage, change of density, morphology and decrease of specific surface area. Unfortunately, no systematic data on bio-aerogel aging is available in literature. The mechanical properties of bio-aerogels should also depend on “aging” time. Ideally, the sample storage and characterisation should be performed in controlled temperature and humidity environment, and sample “age” (time from drying to analysis) reported.

## 7. State of the Art of Bio-Aerogel Particles

A detailed survey shows that the following polysaccharides were used to make aerogel particles, as summarized in Table 1.

### 7.1. Agar Aerogel Particles

Agar is obtained from red algae and consists of two polymers, namely agarose and agaropectin. Agarose is a linear polymer, made of two repeating units of agarobiose, which is a disaccharide made up of D-galactose and 3,6-anhydro-L-galactopyranose. Agaropectin is a mixture of smaller molecules made of alternating units of D-galactose and L-galactose modified with acidic side groups. It is agarose that is responsible for structural transitions and thermotropic gel formation; it is used in aerogel production. 

Robitzer et al. [133,134] have prepared agarose aerogel particles by dropping 2 wt % hot solution at 50 °C in a cooled water bath. Upon supercritical drying, the particles undergo volume shrinkage up to 92%. Total pore volume was 7.5 cm^3^/g with mesopores volume 0.3 cm^3^/g and specific surface area was 320 m^2^/g [133,134].

### 7.2. Alginate Aerogel Particles

**Figure 8:**
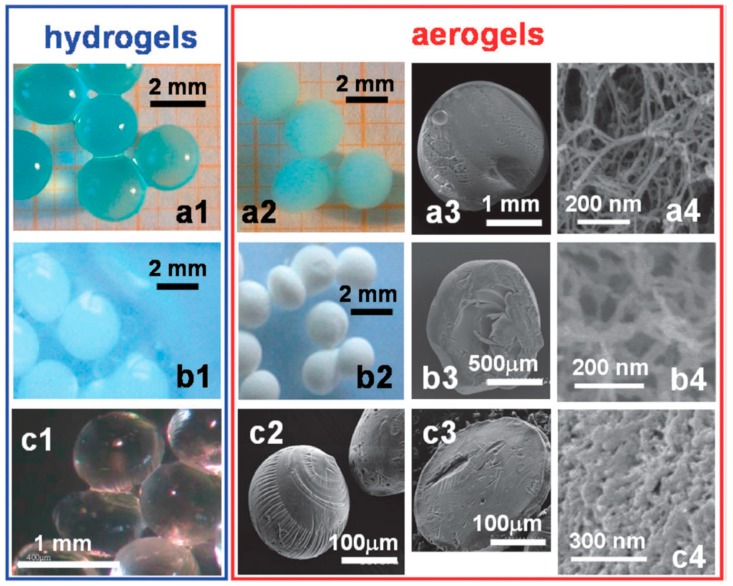
Optical and scanning electron microscopy (SEM) images of hydrogel state (**first column**) and aerogel spheres (**second column**) after drying and of cross-sections of aerogel spheres (**third** and **fourth columns**) of Cu-alginate (**row a**), chitosan (**row b**) and carrageenan (**row c**); reproduced with permission from reference [35].

Commercial grade sodium alginate with a concentration of guluronic acid 67% and mannuronic acid 33% was mostly employed in alginate-based aerogel particles preparation due to high chemical affinity of guluronic acid to di-cations. Quignard and her co-workers have prepared alginate aerogel particles with the syringe dropping method. Most commonly, the solution of sodium alginate is added at room temperature to a stirred solution of calcium chloride or copper chloride (Figure 8a) [35,133,134,158,159,160].

Alginic acid gels can be prepared by protonating the droplets of sodium salt of alginate solution with an aqueous acidic (1 M of HCl) solution [133,134]. This reversing pH method provides nanofibrillar microstructures similar to calcium alginate gel particles. After drying, the volume shrinkage was observed to be 22% and specific surface area was in the range 375–390 m^2^/g [133,134].

The production of alginate gel micro-particles with the emulsion-gelation method has been used in numerous studies for encapsulation of active compound in alginate gel micro-particle [192,193] or to be further processed to aerogel micro-particle (see Table 2) [161,190].

Gelation of the alginate droplet after the emulsification step have been carried out according to the diffusion [194] and the internal setting methods [195].

With the diffusion method, the alginate solution is emulsified in oil and when the desired droplet size distribution is reached, a solution of calcium chloride is gradually added. Even though this method produced gelled micro-particles in the 40 µm range, they were not spherical nor had regular shapes [161].

The internal setting is the most widely used method in the literature for alginate gel micro-particle production and was reviewed by Reis et al. [196]. This method uses an insoluble calcium salt (e.g., CaCO_3_) dispersed in the alginate solution as a vector for calcium ion. The alginate solution and calcium salt mixtures are emulsified and the gelation is triggered via a pH reduction that solubilizes the salt and frees the calcium ion that will form the calcium alginate gel. The most common pH reduction strategy consists of adding acetic acid saturated oil to the emulsion, thus yielding the alginate gel micro-particles.

Instead of adding the acid in the oil, Alnaief et al. [161] showed that it was possible to use (Glucono-δ-lactone) GDL to achieve similar results. In that case, GDL was added to an alginate solution and calcium carbonate mixture before the emulsification. In the presence of water GDL slowly hydrolyses to form gluconic acid, gradually lowering the pH and dissolving the calcium carbonate with time. The characteristics of aerogels are given in Table 2.

The further processing of the alginate gel micro-particle to aerogel micro-particle (solvent exchange and supercritical drying) was achieved by Alnaief et al. and García-González et al. yielding aerogel micro-particles with large surface area and large pore volume as illustrated in the Table 2.

### 7.3. Cellulose Aerogel Particles

Cellulose is a linear polysaccharide composed of D-glucose repeating unit linked by ß(1→4) bonding. Its dissolution in common solvents is difficult because of numerous hydrogen intra- and intermolecular bonds. Typical cellulose solvents are LiCl/dimethylacetamide, N-methylmorpholine-N-oxide monohydrate, (7–9)% NaOH/water, Cu/ethylenediamine, NH_3_/SO_2_/DMSO, molten salt hydrates and ionic liquids [113,197,198,199,200,201,202,203]. Two main ways of making cellulose aerogels via drying under supercritical conditions are known. One corresponds to cellulose dissolution in a direct solvent, like those mentioned above, resulting in cellulose II aerogels. The other is to use cellulose so-called nanofibres, that is, either bacterial cellulose or nanofibrillated cellulose (NFC). Such NFCs are prepared via mechanical disintegration of native cellulose, accompanied quite often by an enzymatic and/or chemical treatment. These materials are initially in the form of a continuous “non-woven” network of cellulose I nanofibers filled with water. Both ways were used to make cellulose aerogel beads.

The production of cellulose aerogel beads have been described for the first time in 1988 [29], but materials were termed porous cellulose particles. They were prepared by dispersing cellulose xanthate (viscose) into the coagulation bath containing alcohol and non-ionic surfactants at temperature range 30–70 °C. Cellulose regeneration was achieved using a neutralization (acidic) medium. In other words, cellulose xanthate was chemically converted to cellulose undergoing hydrolysis reaction in acidic medium. In addition to cellulose-based aerogel particles preparation, cellulose xanthate was used for crosslinking and chemical modification reactions. The obtained size of particles was less than 500 µm. The cellulose-based wet particles were solvent exchanged and dried using supercritical CO_2_. Cellulose crystallinity in aerogels was in the range 23–46%. The crystalline domain phase was composed of type cellulose II phase and an amorphous phase. The pore diameter was in the range between 6 nm to 1 µm.

**Figure 9:**
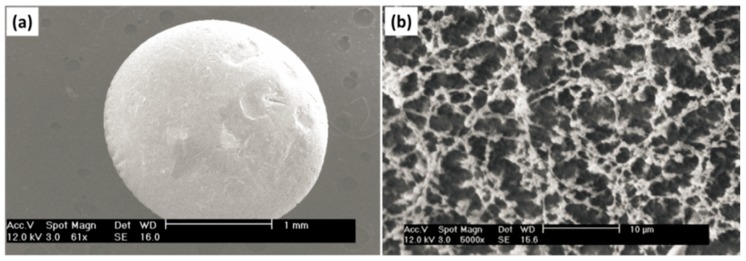
SEM micrographs of cellulose aerogel prepared from 5%Avicel/8%NaOH/water solutions coagulated in 10% H_2_SO_4_ bath: (**a**) cellulose aerogel bead and (**b**) its cross-section. The pictures were adapted from the Thesis of Dr. R. Gavillon, CEMEF, Mines Paris Tech; reproduced with permission from Reference [151].

Alternatively, cellulose aerogel particles were prepared with the JetCutter method. Cellulose was dissolved in 8%NaOH/water and jet-cut into H_2_SO_4_ solution. Wet particles were washed from NaOH in acidic aqueous solution, water replaced by acetone and finally they were dried with supercritical CO_2_ [151]. The SEM images show the particle and the internal morphology (Figure 9).

The same jet-cutting technology was used to prepare cellulose aerogel particles using a cellulose derivative, namely preparing a cellulose carbamate which was dissolved in NaOH/water [155]. Cellulose was regenerated from cellulose carbamate either by thermal or chemical treatment in an acid-salt bath. Particles of about 0.5 mm were formed and dried using supercritical CO_2_. Depending on the polymer concentration the specific surface area varied from 350 to 540 m^2^/g [155].

**Figure 10:**
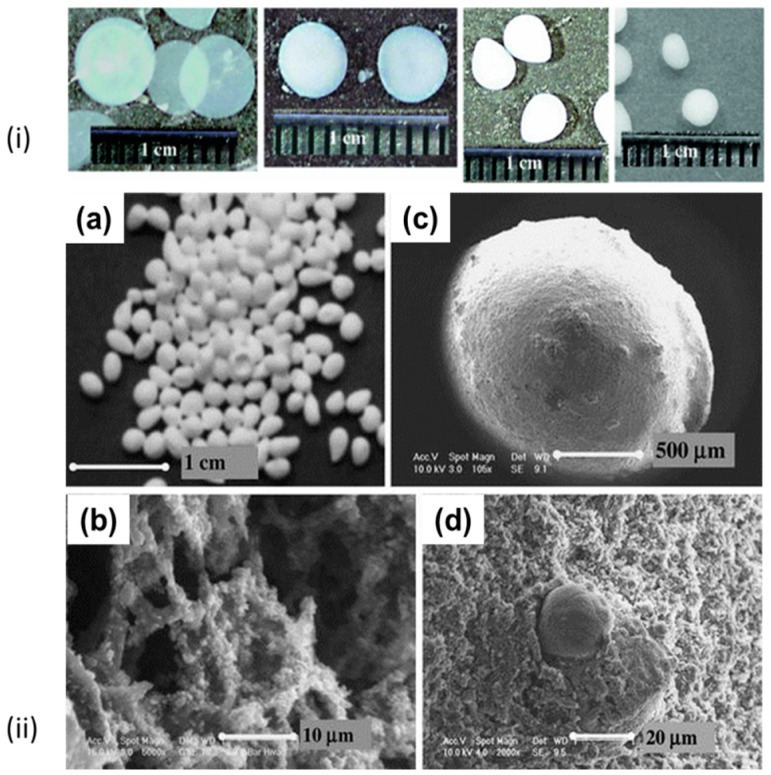
Wet and dry (aerogels) cellulose particles: (**i**) top row—the photographic images of wet cellulose particles (coagulated cellulose in water) of various shapes and (**ii**) bottom row—photographic image of TiO_2_-encapsulated cellulose aerogel (**a**), its morphology by SEM (**b**) and SEM images of the morphology of iron-encapsulated cellulose aerogel particles (**c**,**d**); reproduced with permission from reference [142].

Sescousse et al. reported on using NaOH/water as cellulose solvent and making particles via non-solvent induced phase separation pathway: by dropping cellulose solution in water, exchanging water by acetone and drying with supercritical CO_2_. They showed that the shape of the particles can vary from very flat plates to spheres (Figure 10—top row) [142]. The influence of the preparation conditions on the shape of the particles, like cellulose concentration, delay time, bath temperature, and the distance between the pipette and the bath surface was discussed. They were also able to incorporate various inorganic powders into cellulose particles and thus to prepare organic-inorganic aerogel particles (Figure 10—bottom row).

Similar solvent, 7%NaOH/12%urea/water, was used to make cellulose particles via dropping method and particles size, shape and surface area were modified by coagulation bath conditions [146]. After supercritical drying, their volume in the wet state was 8–20 mm^3^ and specific surface area was 330–470 m^2^/g. The same solvent was used to make particles via emulsion method; in wet state their diameter varied from few microns to 1 mm; however, these particles were freeze dried resulting in much lower specific surface area around 16 m^2^/g [150].

Another additive, ZnO, was used for enhancing the dissolution of cellulose in the mixture of NaOH/urea/water [144]. Urea and ZnO are usually employed to delay cellulose gelation and improve dissolution. The cellulose solution prepared from the solvent mixture, NaOH/ZnO/urea/water, was dropped in an acidic aqueous medium. The studies showed that the addition of 0.5 wt % of ZnO to the NaOH/urea/water mixture can effectively improve the specific surface area and microstructure of cellulose aerogel particles (for example, BET specific surface area was 407 m^2^/g).

Omura et al. [204] have reported the spongy cellulose particles which can be used for the encapsulation of hydrophilic and hydrophobic fluorescent molecules. Cellulose was dissolved in ionic liquid, 1-butyl-3-methylimidazolium chloride and N,N-dimethylformamide. The particles were prepared by dispersing the cellulose solution in n-hexane containing a dissolved surfactant in order to make spherical droplets. The particles were then washed in protic non-solvent medium, 1-butanol. The specific surface area of supercritically dried samples showed a higher value of 371 m^2^/g in comparison with vacuum dried sample (a dense structure having < 1 m^2^/g). 

1-allyl-3-methylimidazolium chloride, an ionic liquid was used as solvent to prepare cellulose particles from cellulose paper wastes [173]. Using a syringe, the cellulose solution was dropped into a water coagulation medium. The specific surface area was varied from 101 to 478 m^2^/g. The authors employed different drying techniques and compared the microstructures. The aerogels showed open porous nanofibrillar structure with high surface area in comparison with oven-dried and air-dried samples. In addition, aerogels showed high loading capacity of dye molecule, curcumin, which is about 0.55 mg/g.

**Figure 11:**
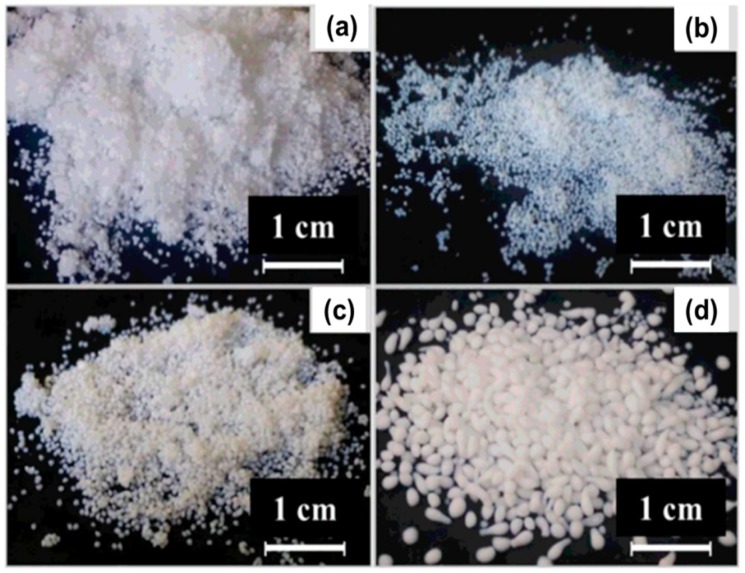
Cellulose aerogel beads from 2 wt % (**a**–**c**) and 3 wt % (**d**) cellulose-[DBNH][CO_2_Et] solutions coagulated in water (**a**), isopropanol (**b**) and ethanol (**c**,**d**); reproduced with permission from reference [175].

Another ionic liquid, 1,5-diazabicyclo[4.3.0]non-5-enium propionate ([DBNH][CO_2_Et]) and Jet-cutting methods was used recently to produce cellulose aerogel beads (Figure 11) [175]. The importance of the rheological properties of cellulose solutions in jet-cutting was demonstrated. The size of particles varied from 0.5 to 1.8 mm depending on cellulose concentration, density was 0.04–0.07 g/cm^3^ and specific surface area was 240–300 m^2^/g.

**Figure 12:**
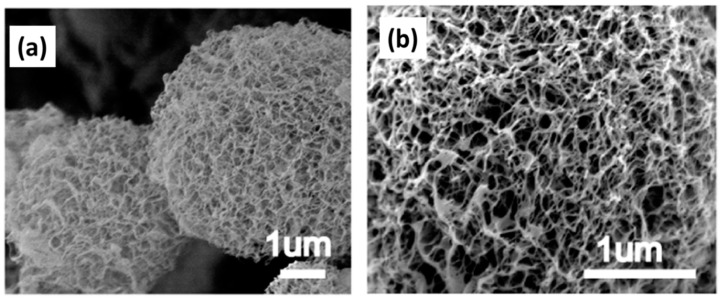
SEM images of TEMPO oxidized cellulose nanofibril-based aerogel microspheres (**a**) prepared by freeze drying method and the particles morphology under the higher magnification (**b**); reproduced with permission from reference [95].

A special case of highly porous nanofibrillated cellulose particles should be mentioned: using a combination of atomization and freeze drying, named spray-freeze drying, the production of finely distributed cellulose nanofibril aerogels was reported [94,95]. The TEMPO ((2,2,6,6-Tetramethylpiperidin-1-yl)oxyl) oxidized cellulose nanofibrils were crosslinked with Kymene and then the suspension was sprayed at 40 MPa pressure through a 1 mm inner diameter steel nozzle into the liquid nitrogen bath. After freeze drying, the crosslinked cellulose nanofibrils aerogels showed high specific surface area 390 m^2^/g with a cellulose nanofibril concentration of 0.6% [94]. The size of aerogel particles was in the range of 2–7 µm (Figure 12) [95].

Beamount et al. [174] produced cellulose II particles by dispersing enzymatically pre-treated TENCEL^®^ gel (coagulated in water wet cellulose from cellulose-NMMO solution, Lyocell process) using a high pressure homogenizer. The latter broke TENCEL^®^ gel into particles. The enzymatic treatment significantly reduced cellulose molecular weight down to 19 kg/mol. The particles were then either freeze-dried from water or water was exchanged to tert-butanol and then freeze-dried. This method provides the particles with irregular shape having particle size in the range between 0.5 to 10 µm. After freeze drying from tert-butanol medium, the particles showed the continuous open porous structure having nanofibrillar network with high specific surface area of 298 m^2^/g. The particles were termed cryogels by the authors. The particles obtained by freeze-drying from water showed sheet-like morphology, as in [140,163], with no considerable specific surface area (10 m^2^/g).

### 7.4. Chitin and Chitosan Aerogel Particles

Chitin is a linear polysaccharide having N-acetyl-D-glucosamine repeating units linked by ß(1→4) bonding. The copolymer containing N-acetyl-glucosamine and N-glucosamine units are also called chitin when the polymer chain has more than 50% N-acetyl-D-glucosamine units. Chitosan is one of the most important derivatives of chitin with the degree of deacetylation of N-acetyl-glucosamine units higher than 50%. While chitin is very difficult to dissolve, chitosan offers water solubility under mild acidic condition by protonation of amine functional groups (pK_a_ = 6.3). The increase of pH above pKa leads to polymer micro-phase separation, similar to that when placing cellulose solution in water, and a “wet” chitosan network is formed.

In the literature only one reference can be found for chitin aerogel particle production [153]. Silva et al. [153] have reported the production of chitin and chitin-silica composite aerogel particles by employing a non-solvent phase separation method in which ionic liquid, 1-butyl-3-methylimidazolium acetate, was used as the solvent medium to dissolve the chitin powder. Particles were made by simple syringe dropping method in ethanol. Though supercritical CO_2_ drying was employed, the porosity was reported to be rather low, 66–67.3% and pore size distribution was in the range 257–359 µm [153].

Quignard, Valentin and co-workers [35,67,133,134,135,158] used squid-pen and crab-shell chitosan with a high degree of amine groups to prepare chitosan aerogel particles. Chitosan was dissolved in acetic acid and the solution was dropped in an aqueous solution of sodium hydroxide through syringe needle with a nozzle diameter of 0.8 mm. Gelation occurred immediately at the droplet surface. Aerogel particles preparation then proceeds as usual: washing with water, exchange with ethanol and drying with supercritical carbon dioxide. The aerogel particles had a fibrillar microstructure (see Figure 8b), exhibited a volumetric shrinkage upon drying of around 50% and specific surface area from 150 to 300 m^2^/g.

Recently, León et al. has demonstrated supercritical drying of chitosan-tripolyphosphate nanoparticles [185]. The nanoparticles diameter was in the range of 70 to 180 nm (polydispersity index = 0.36). Unfortunately, the supercritically dried nanoparticles (aerogels) showed low specific surface area of around 11 m^2^/g; freeze-dried chitosan-tripolyphosphate nanoparticles had even lower specific surface area.

Chitosan was used to prepare organic-inorganic hybrid aerogel microparticles. To achieve this, first the inorganic components are hybridized with the chitosan polymer using either a co-gelation or a post gelation method [66,176,177,178,179,180,181,182,184]. Chitosan-montmorillonite clay hybrid aerogels microparticles were recently reported [176,177]. In these the organic and inorganic components are well mixed together before the gelation occurred. Montmorillonite belongs to the class of sodium-aluminium layer silicates, is quite abundant in nature (part of bentonite). The layer structure and its hydrophilicity make it interesting for nanoreinforcment of certain polymers. A hybrid with chitosan is achieved by mixing an acidic aqueous solution of chitosan with sodium salt of montmorillonite. Being a cationic polymer in the acidic medium, the ammonium ions of chitosan can intercalate the layers of montmorillonite and thus increase the interlayer spacing. When this acidic dispersion is dropped into an alkaline solution, the gel microparticles of chitosan-montmorillonite are formed. Washing and supercritical drying of the hydrogels yield aerogels. These hybrid aerogels have low specific surface area in comparison with chitosan aerogels. However, these hybrid aerogels showed enhanced thermal properties with the degradation delay in the order of chitosan < chitosan-graphene oxide < chitosan-clay aerogels [176,177,183].

In post gelation method, chitosan gel particles prepared by dropping method in alkaline solution are utilized to form organic-inorganic composite microspheres by impregnation of the second phase into chitosan network. Chitosan gel particles are dipped in the precursors of metal oxide sol for 12 to 48 h. Sol-gel process occurred in the organic matrix of microparticles to form composite materials. After washing, the particles are dried by supercritical CO_2_ yielding organic-inorganic composite aerogel microparticles [66,178,179,180,181,182].

### 7.5. Κ-Carrageenan Aerogel Particles

Κ-carrageenan is a sulphated polysaccharide belonging to carrageenan family. Quignard and her co-workers used syringe dropping method to prepare κ-carrageenan aerogel particles [35,133,134,186]. The hot solution of κ-carrageenan was taken in a thermostated syringe and dropped into a cold saline (KCl) solution. SEM image in Figure 8c shows the very closely packed fibrillar network. The aerogels showed 95% of volume shrinkage and specific surface area of about 200 m^2^/g. The volume shrinkage can be reduced if the specific ions are chosen to be potent helix stabilizers [205]. The reports also demonstrated that the massive volume shrinkage can be limited to almost no shrinkage when the nanofibers of κ-carrageenan were impregnated with silica sol particles [186].

Alnaief et al. [187] have prepared κ-carrageenan aerogel particles by emulsion method in which the influences of parameters such as specific cations and anions have been studied. Using specific ions, potassium cation and carbonate anion was shown to produce better aerogel textural properties in comparison with other cations (Ca^2+^ and Al^3+^) and anions (Cl^−^ and I^−^). Using potassium carbonate as specific ions for gelation, the results showed a specific surface area of 167 m^2^/g, a pore volume of about 0.54 cm^3^/g and about 13 nm as a pore diameter [187].

### 7.6. Pectin Aerogel Particles

Pectin is linear polysaccharide mainly consisting of galacturonic acid units which are connected via α-(1-4) bonds. Three types of pectin are to be differentiated: (1) low methoxyl (LM) pectin, where less than 50% of galacturonic acid groups are esterified with methyl groups; (2) high methoxyl (HM) pectin, where more than 50% of existing galacturonic acid groups are esterified with methyl groups; and (3) amidated pectin, where acid groups are partly amidated. Gelling properties highly depend on the ratio of esterified and amidated acid groups [206].

Veronovski et al. [188] prepared multilayer amidated LM pectin aerogel particles via ionotropic gelation by dropping 2% pectin solutions through a needle (0.8 mm in diameter) into calcium chloride (CaCl_2_) solution. After gelation, obtained hydrogel particles were dropped into a 1% pectin solution to form membranes (layers) around the core, and again were gelled with CaCl_2_. Three membranes were produced around the core. Solvent was exchanged in successive ethanol-water baths and particles were dried with supercritical CO_2_. These aerogel particles were loaded with theophylline and nicotinic acid; the drugs were directly dissolved in the initial pectin solution and the same procedure of multi-layer preparation was repeated. Multilayer pectin aerogel particles with diameter of 8.0 and 9.8 mm, depending on the source of pectin (apple and citrus, respectively), were produced. Specific surface area of obtained cores and membranes varied from 469 to 593 m^2^/g depending on source of pectin and initial pectin concentration. The release of both theophylline and nicotinic acid from citrus pectin turned out to be slower than that from apple pectin, around 7 h versus 1.5 h, respectively. It was shown that release is controlled by swelling and dissolution of pectin matrix.

De Cicco et al. [74] combined amidated LM pectin with alginate to produce core-shell aerogel particles. A core of pectin was covered with a layer of alginate using the co-axial prilling method. Ionotropic gelation was performed via diffusion method in an ethanolic CaCl_2_ solution. Smooth, spherical aerogel core-shell particles with diameters between 3.23 and 3.26 mm were obtained. The particles were loaded with doxycycline hyclate which was dissolved in pectin solution. The density of aerogel particles was around 0.3 g/cm^3^.

**Figure 13:**
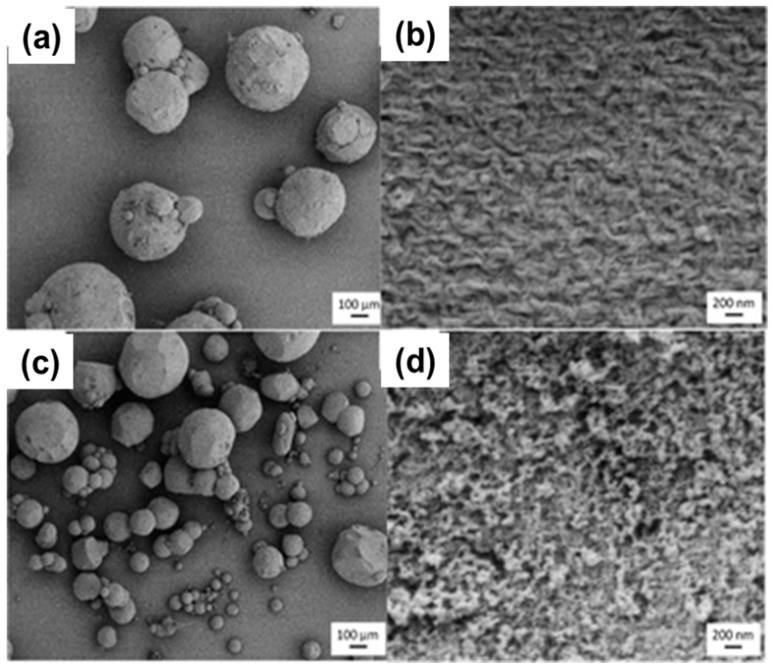
Pectin (**a**,**b**) and pectin-maghemite aerogel particles (**c**,**d**); reproduced with permission from reference [189].

García-Gonzáles et al. [189] produced HM pectin aerogel microspheres via emulsification in oil at 313 K for 30 min followed by coagulation in ethanol. Highly spherical pectin aerogel particles with specific surface area of 440–480 m^2^/g and particle diameter between 100 and 2000 μm, depending on the oil:water ratio, were obtained. Pectin solution was mixed with maghemite nanoparticles and magnetic pectin aerogels were produced (Figure 13). The incorporation of nanoparticles slightly increased specific surface area and aerogel density. The same production method was used later by García-Gonzáles et al. [190] to produce HM pectin aerogel microspheres with mean mesopore diameters 14–18 nm. Hereby, pectin aerogel microspheres with specific surface area of 379 m^2^/g and mean particle diameter before drying of 498 μm were obtained.

### 7.7. Starch Aerogel Particles

As described in Section 3.1. “Gelation of polysaccharide solutions,” starch granules swell in hot water and gelatinization occurs. Retrograded starch gels can then be the precursor for making starch aerogels.

The first starch based aerogels, called “microcellular foams,” were reported by Glenn and Irving in 1995 [207]. The density was around 0.22–0.23 g/cm^3^. More than a decade later, starch aerogels in the shape of cylinders were prepared via dissolution-retrogradation solvent exchange-supercritical CO_2_ drying route and suggested to be used as matrices for drug delivery applications [195]. For potato-based aerogels the density was rather high, around 0.3–0.45 g/cm^3^ and specific surface area was rather low, around 70–90 m^2^/g [195]. Later publications report aerogels from wheat starch with density varying from 0.05–0.3 g/cm^3^ and specific surface area around 50–60 m^2^/g [208]; low density and high specific surface area was reported for high amylose corn starch: 0.14 g/cm^3^ and 254 m^2^/g, respectively [49].

Starch aerogel particles were prepared from corn and pea starches via emulsion-gelation method: first, by making starch solution/oil emulsion at 393 K and then retrograding starch in the droplets [190,209] at 318 K. Aerogel particles were of 400–800 µm in diameter with densities of 0.1–0.25 g/cm³ and specific surface area in the range of 100–240 m^2^/g. These particles were used as matrices to deliver ketoprofen and benzoic acid which were loaded in starch matrix under supercritical CO_2_.

Starch aerogel particles with high amylose-fatty acid complexes were reported by Kenar et al. [191]. The retrograding property of amylose was controlled by blending starch with the sodium palmitate where the polyelectrolyte property of amylose-complexed sodium palmitate prevents the gel formation. The hydrogels were prepared by dropping the solution of amylose corn starch-sodium palmitate using syringe into the hydrochloric acid solution. Specific surface area of aerogels was in the range between 313 and 361 m^2^/g.

## 8. Applications of Polysaccharide Aerogel Particles

Application of aerogels and in particular polysaccharide aerogel particles is a subject of growing interest and stimulating research. Progress over the last two decades has been recently summarized in several review articles [14,19,24,210,211]. Here we intend to highlight those applications where the use of particles plays a crucial role. Polysaccharide aerogel beads with their physical, chemical and functional properties can be used in applications such as separation techniques, catalysis or as carriers for drug delivery.

Perhaps the most studied application area suggested for polysaccharide aerogels is drug delivery. Pioneered in early 2000’s for silica aerogels [212,213], the use of aerogels as carrier matrices for drugs had expanded over the years to include many new aerogel classes, mainly biopolymers [19]. Being largely mesoporous solids, aerogels can accommodate drugs in the amorphous state suppressing re-crystallization [214]. This feature along with the high specific surface area and rapid pore collapse upon contact with liquid media gives rise to unusually fast drug release. The enhanced drug release has clearly been shown for classical silica aerogels [215], whereas for the case of biopolymer, other factors such as pH-dependent swelling and degradation, also contribute to the overall release kinetics [188,190]. Furthermore, release profiles vary with the particle size—a useful effect for the modulation of the release kinetics but often overlooked when reporting such kinetic data. As discussed in the previous sections, a variety of particle generation and gelation techniques are reported to date. They can potentially cover the size requirements for a specific administration route allowing for a reasonably high throughput. Oral [190,216,217], mucosal [218], and most recently pulmonary [219] drug delivery routes have been exemplified in the literature. Furthermore, thanks to high pore volume and swelling ability both pristine and drug-loaded polysaccharide aerogel particles have been suggested as superabsorbent and for wound healing applications [56,74].

Another active line of research is the use of polysaccharide aerogels as advanced food materials [220]. Edibility, renewability, sustainability and relatively low cost of polysaccharides make them an attractive starting material for functional food. When used as food, polysaccharide aerogels could function as dietary fibre (e.g., cellulose) or as a source of energy (e.g., starch). Analogously to drug carriers, aerogels may serve as a hosting matrix for active compounds and nutraceuticals (e.g., vitamins, microelements), see [221]. They can also be utilized as mechanical support structures in food packaging and water absorbents in active packaging [220].

Beyond that, polysaccharide-derived aerogels demonstrate a thermal conductivity in the range of 16–22 mW/m·K [24], which is lower than for conventional biomass-derived materials and comparable with well-studied silica-based aerogels. Although the explicit use of polysaccharide aerogel particles in thermal insulation applications is unknown, to the best of our knowledge, particulate silica aerogels have widely been investigated for this purpose [222,223]. With the newest advances such as hydrophobization [14] and reinforcement [224] methods as well as post-functionalization (e.g., coating, see [225,226]) and fast supercritical drying [227,228,229] we may expect this area to expand in importance in the upcoming years.

Further application fields embrace environmental remediation and catalysis, both of them have been comprehensively reviewed very recently [210,211]. Functionalized nanofibrillar network of polysaccharide aerogel beads have been utilized as promising reusable filter materials for the applications in environment control system [172,230,231]. High specific surface area of nano-network and chelating functional groups of polysaccharide aerogels offer hosting property and they can be utilized for catalysis applications [65,66,67,68]. For instance, it was demonstrated that alginate and chitosan aerogels can act as supports in heterogeneous catalysts as the carboxylate anion in the alginate and amine groups in chitosan aerogels can offer the guest binding properties, coordinating the metal nanoparticles or complexes [65,66].

## 9. Conclusions and Perspectives on the Potential Scale-up Production of Bio-Aerogel Particles

Table 3 shows an overview of the processes applicable for the production of gel particles which can be later solvent exchanged and dried to obtain aerogels.

The easiest way to prepare particles is the simple dropping process: particles are made by dropping a solution into a gelation bath using a syringe or pipette. This elegant process facilitates an easy way to study and understand in depth the influence of various parameters, like biopolymer concentration, type of solvent, solution viscosity and bath parameters on the formation of particles, meaning especially their size, shape and morphology. Whenever a new system is prepared the application of this method is inevitable and builds the background for further up-scaling steps. Going to a larger scale, the “dropping” method must be replaced by other methods like spraying, jet-cutting or atomization. Then, of course, the processing parameters have to be adjusted. The jet-cutting process has been successfully employed for the preparation of micrometre and millimetre sized particles. The jet-cutting technique is recommended for polysaccharide solutions in terms of large scale productivity of particles. Production rates of up to hundreds of kilograms of gel particles per hour seem adequate considering the number of particles needed for a pilot plant. It should be noted that the JetCutter technology for making gel beads is already approved for large scale by geniaLab^®^. Although the JetCutter technology looks as if it is the appropriate choice for biopolymer solutions of high viscosity, it has a drawback for solutions of low viscosities in the range of 200–300 mPa.s: the spray losses increase with decreasing viscosity. In the spraying atomization small particles can be prepared, however the viscosity of the solutions is a main issue along with the pipe blockage due to the occasional gelation therein. Spraying atomization is widely used in the pharmaceutical industry so that the scalability is already proved.

**Figure 14:**
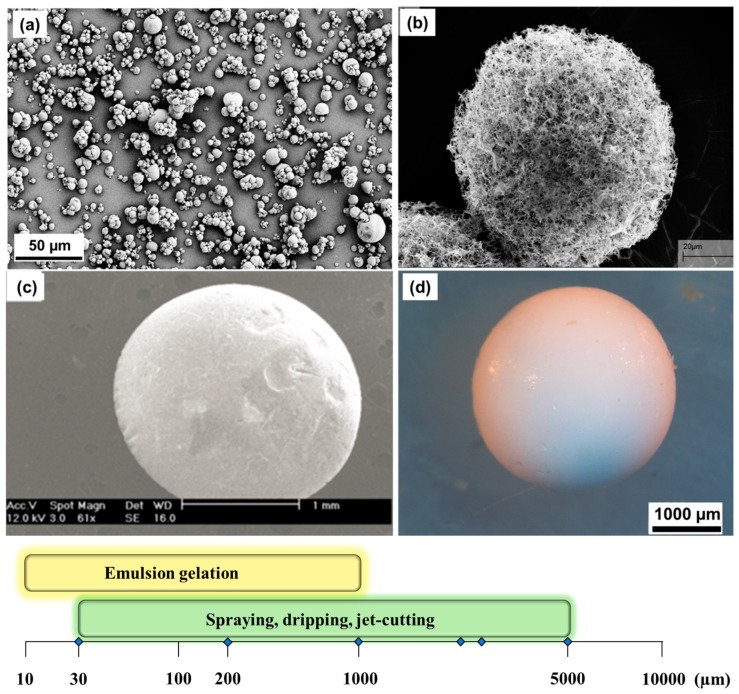
Comparison of aerogel particles prepared by different techniques. Scanning electron microscopy images of particles prepared by emulsion (**a**), spraying, image reproduced with permission from [94] (**b**), jet-cutting (JetCutter), image reproduced with permission from [151] (**c**) and light microscopy image of aerogel particles by syringe dropping method (**d**). Sketch at the bottom showing the range of particle sizes prepared by employing suitable preparation methods.

In emulsion gelation method, agitation or stirring can create steady-state conditions, such that sol-droplets are kept in suspension indefinitely, or as long as needed to complete the gelation reaction within the sol-droplets. The production of particle sizes of 1 to 1500 µm using the emulsion gelation method can easily be realized. Spherical particles can generally be obtained. As in the case of the simple dropping method, the influence of process parameters has to be carefully examined using physical and/or design of experiments approaches. Prior to any process design it is necessary to acquire physico-chemical data of the systems to be used, like densities, viscosities, partition coefficients, gelation kinetics and their temperature dependence. Therefore, particles of different sizes can be produced applying the suitable method, as summarized in Figure 14. Therefore, we are optimistic that in the very near future the aerogel production in form of particles would make a significant progress. This topic is extensively studied in the EU project NanoHybrids.

## Figures and Tables

**Table 1:** Bio-aerogels in form of particles produced by different gelation methods and dried with supercritical CO_2_.

Biopolymer	Gelation Process	Particles Preparation Method
Agar	Thermotropic	Syringe dropping method [133,134].
Alginate/Alginic acid	Ionotropic and pH	Syringe dropping method [35,133,134,158,159,160]; Emulsion method [161]; Prilling method [74]; JetCutter [172].
Cellulose	Non-solvent-induced phase separation	Syringe dropping method [29,142,144,146,151,155,173]; Atomization method [94,95]; dispersion of wet-coagulated cellulose [174]; JetCutter [151,175];
Chitin	Non-solvent-induced phase separation	Syringe dropping method [153].
Chitosan	Ionotropic and pH	Syringe dropping method [35,66,67,133,134,135,158,176,177,178,179,180,181,182,183,184]; Dispersion of liquids method [185]; JetCutter [172].
Κ-carrageenan	Thermotropic and ionotropic	Syringe dropping method [35,133,134,186]; Emulsion method [187].
Pectin	Thermotropic and ionotropic	Syringe dropping method [188]; Prilling method [74]; Emulsion method [189,190]; JetCutter [172].
Starch	Thermotropic	Syringe dropping method [191]; Emulsion method [19,190].

**Table 2:** Physical properties of aerogels of alginate particles obtained from references [161,190].

Gelation Method	Particle Size, µm	Surface Area (BET), m²/g	Mesopore Volume (BJH), cm³/g	Reference
Diffusion method (calcium chloride)	~40	394 ± 71	10 ± 2	[161]
Internal setting with acetic acid	150–1400	590 ± 80	15 ± 2	[161]
Internal setting with GDL	~40	469 ± 54	13 ± 3	[161]
Internal setting with acetic acid	116 ± 6	524 ± 26	-	[190]

**Table 3:** An overview of the processes applicable for production of polysaccharide gel particles (after drying, aerogels can be produced).

	Dropping	JetCutter	Spraying Atomization	Emulsion Gelation
Particle size	0.2–10 mm	0.2–10 mm	1–500 µm	10–500 µm
Scale of the process (productivity)	1 g/min (10 nozzle system) ^a^	Technical scale (several kg/h)	Productivity depends on the nozzle	Technical scale possible
Needs of additives	No	No	No	Surfactants and oil
Advantages	Very simple	Versatile systems, simple, commercially available apparatus; monodispersed particles	Different sizes possible depending on the nozzle	Simple apparatus Particle size can be regulated by stirring and surfactants
Limitations	Limited productivity	Not suitable to produce small particles (<200 µm); limited processing window in terms of the rheological properties of solutions	Clogging, polydispersed particles	Washing from oil; Polydispersed particles

^a^ the droplets are freely falling under the gravitation force; viscosity of fluid is 1.8 Pa.s at a shear rate of 1 s^−1^.
